# The Mere Co-Presence: Synchronization of Autonomic Signals and Emotional Responses across Co-Present Individuals Not Engaged in Direct Interaction

**DOI:** 10.1371/journal.pone.0125804

**Published:** 2015-05-27

**Authors:** Yulia Golland, Yossi Arzouan, Nava Levit-Binnun

**Affiliations:** Sagol Center for Brain and Mind, Baruch Ivcher School of Psychology, Interdisciplinary Center (IDC), Herzliya, Israel; Liaoning Normal University, CHINA

## Abstract

Existing evidence suggests that in social contexts individuals become coupled in their emotions and behaviors. Furthermore, recent biological studies demonstrate that the physiological signals of interacting individuals become coupled as well, exhibiting temporally synchronized response patterns. However, it is yet unknown whether people can shape each other's responses without the direct, face-to-face interaction. Here we investigated whether the convergence of physiological and emotional states can occur among “merely co-present” individuals, without direct interactional exchanges. To this end, we measured continuous autonomic signals and collected emotional responses of participants who watched emotional movies together, seated side-by-side. We found that the autonomic signals of co-present participants were idiosyncratically synchronized and that the degree of this synchronization was correlated with the convergence of their emotional responses. These findings suggest that moment-to-moment emotional transmissions, resulting in shared emotional experiences, can occur in the absence of direct communication and are mediated by autonomic synchronization.

## Introduction

It has been widely demonstrated that in social contexts people significantly influence and shape each other’s inner states and behaviors. Moreover, during direct interactions individuals have a strong tendency to synchronize with their interacting partners in their emotions, expressions and body movements [1–6]. For example, studies demonstrated that people automatically mimic facial expressions and experience corresponding emotional states, both when they view emotional expressions on a computer screen and when they face real partners in dyadic interactional setups [7–10]. Along similar lines, people engaged in conversation synchronize their body sway [6] and their voices converge in frequency [11]. Thus, one of the central themes emerging in studies of social interactions is that individuals’ behavioral and emotional states become tightly coupled as a result of interaction [12–14].

Remarkably, a rapidly growing body of research suggests that individuals can become coupled not only in their behavioral responses but also in their physiological activity [15–17].A series of recent studies demonstrated that the neural signals and the autonomic measures of individuals during interactional exchanges become temporally synchronized at timescales of seconds [16–18]. For example, imaging studies that investigated the information flow between two brains found that when individuals communicated information to each other using hand gestures [19], facial expressions [20] or verbal story, their brain activity became synchronized in a functionally selective manner. Brain-to-brain coupling was also found during ongoing interactions such as reciprocal gaze exchanges, verbal communication [21] and spontaneous motor synchronization [22].

Similar interpersonal interdependencies in continuous response patterns were observed in the emotional domain, using autonomic nervous system measures[23–25], which are tightly linked to individuals’ emotional experiences [26,27]. In particular, psychophysiological research showed that emotionally laden interactions, such as marital conflicts, gaze exchanges and empathic processes involve enhanced synchronization of autonomic signals (e.g. heart rate, electrodermal activity) across interacting individuals [24,28–31].

Recent models suggest that the observed propensity of interacting individuals to become coupled in their behavioral and physiological responses results from the biologically mediated tendency to adapt to incoming social information [15,32,33]. Specifically, these models emphasize that during interaction individuals rapidly and continuously exchange information via verbal and non-verbal routes [12,34]. In this process, the information coming from another individual is mapped onto one’s own body and brain, resulting in shared behavioral and physiological states [35]. Accordingly, the physiological coupling, that is the alignment of physiological activity in interacting individuals, marks the contagious spread of information between people which promotes shared states of meaning, feelings and intentions [15,16]. It should be noted that although both behavioral and physiological interpersonal synchrony have been suggested as mechanisms of social interaction, the direct evidence linking these two phenomena is still insufficient [16].

Previous research on physiological coupling has mostly focused on explicit communication, in which information was intentionally exchanged between individuals. However, it is still unclear whether the contagious spread of information from one person to another necessitates direct interaction. Indeed, only a handful of behavioral studies have investigated the across-person influences in the “mere co-presence” conditions, in which individuals are physically co-present, but do not engage in direct interactional exchanges. These studies have provided initial evidence that behavioral coupling, such as synchronized body movement [36] and convergent emotional responses [37] can occur unintentionally.

**Fig 1:**
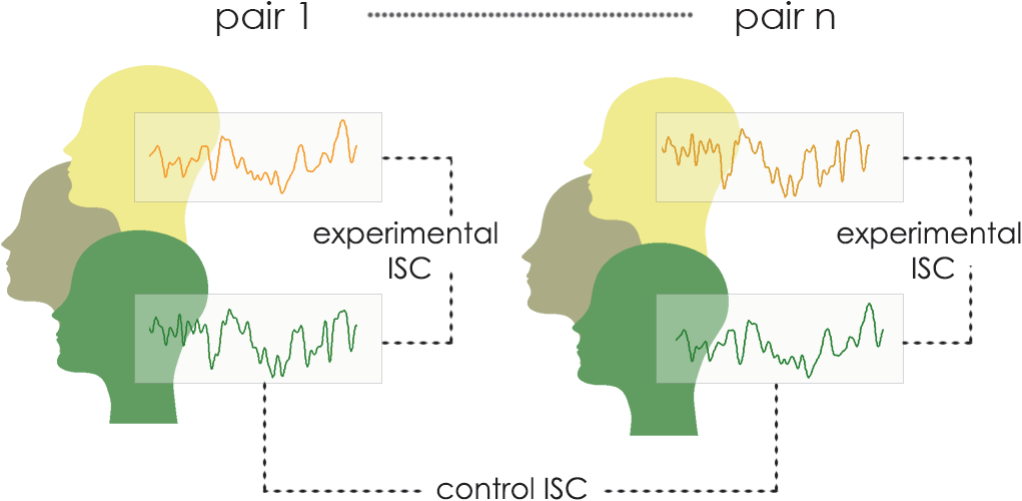
Analysis approach. The effect of co-presence on autonomic synchronization was assessed by comparing experimental and control inter-subject correlation (ISC) of participants’ autonomic signals. Experimental ISC was calculated for participants who viewed the movie together, in the same group. Control ISC was calculated for participants who viewed the movie not together, in different groups.

In the present study we asked whether individuals can distinctively affect each other’s physiological states and emotional experiences in the absence of direct communication. Specifically, we investigated whether merely co-present individuals can become coupled in their autonomic responses and whether this coupling is associated with the convergence of their emotional responses. To this aim, we measured the cardiovascular and electrodermal activity of participants who sat next to one another and watched emotional movies. Participants were positioned at the periphery of each other's visual field: their attention was focused on external emotional stimulus and they did not directly interact with each other. We hypothesized that emotional transmissions would indeed occur in such a minimal social setup, leading to enhanced autonomic synchronization between participants who watched the movies together, compared to participants who watched the movies in different groups (Fig 1). In addition, we hypothesized that participants’ autonomic synchronization would be directly associated with the convergence of their emotional responses to movies, reported after the viewing. In other words, co-present participants who exhibited higher autonomic synchronization with each other would also report more similar emotional responses to the presented movies.

## Materials and Methods

### Participants

We recruited 26 triples of female acquaintances who were psychology undergraduate students and participated in the experiment for course credits. The study was approved by the Interdisciplinary Center research ethics committee (IDC IRB), and written informed consent was obtained after the procedures had been fully explained.

### Experimental Procedure

Pilot testing was performed to identify the optimal experience of co-presence. We noticed that when participants entered the lab (equipped with multiple recording devices) they tended to retract into themselves and reported a mild social experience. To enhance the experience of co-presence we decided to work with triplets rather than couples of people, which appeared to enhance the social nature of the experimental setup.

Upon entering the lab, the three participants were seated side-by-side on a long couch. The participants seated in the middle and far left were hooked to physiological sensors (overall 52 participants). The participant seated at the far right was connected to mock sensors, but no actual recordings were taken due to technical constraints of the measurement devices. Participants were required to refrain from talking and making gross movements throughout the whole experiment.

Two pilot-tested emotional movies were presented in a counterbalanced order. The positive movie consisted of excerpts from the comedy "When Harry Met Sally" (449 sec). The negative movie consisted of excerpts from the horror movie "Paranormal Activity" (434 sec). Each movie was preceded by a 180 sec neutral movie comprising nature scenes, to neutralize participants’ emotional and physiological conditions. Following the neutral movie a brief summary of the emotional movie’s plot was presented for 30 sec.

### Behavioral measures

Following each movie, participants rated the degree of distinct emotions elicited by the movie, using an 11-points Likert-like scale [38]. For the mean level analyses of emotional responses, we conservatively used the pairs' average scores [39].

At the end of the experiment participants provided information about the level of acquaintance with the other participants on an 11-points Likert-like scale, ranging from 0 (don’t know her) to 11 (my best friend).

### Physiological data collection and preprocessing

During the experimental session, continuous physiological measures were recorded at a sample rate of 1 kHz with an integrated system and software package (Mindware Technology, Gahanna, OH). Three measures were obtained: electrocardiogram (ECG), electrodermal activity (EDA), and respiration (not analyzed in this study). Cardiovascular responses were recorded with an ECG amplifier module and disposable snap ECG electrodes using a modified lead II configuration. The heart period (inter-beat interval, or IBI) was assessed using the Mindware HRV 2.16 biosignal processing module by (a) identifying the R–R intervals; and (b) detecting physiologically improbable R–R intervals based on the overall R–R distribution using a validated algorithm [40]. Data were also inspected manually to ensure that R-waves were correctly identified. Data that included more than 10% undefinable Rs was excluded from the analysis. IBI series were transformed to continuous 2 Hz heart rate time-series (HR) using custom software. Electrodermal activity (EDA) was recorded using Beckman electrodes attached to the palmar surface of the middle phalanges of the first and second fingers of the non-dominant hand. Continuous (2 Hz) EDA signals were extracted using Mindware’s EDA 2.1 software. These signals were examined for gross motion artifacts and for detection of non-responsive subjects (failing to exhibit SCR>0.025 μS in at least 10% of the data), which were excluded from the analysis. Finally, linear trends were removed from EDA signals.

### Missing Data

Sample size was determined on the basis of previous studies run in the lab. We aspired to collect N = 25 groups. Our previous studies showed that this N is optimal for relatively stable results, taking into account the high rate of noisy or nonresponsive EDA measures (~25%). One group of participants in our study failed to refrain from talking during the experiment and was therefore excluded from the analysis. Therefore we sampled N = 26 groups. Since the main analysis in this study is dyadic, an exclusion of one of a pair’s member due to unreliable physiological data lead to the exclusion of both members from the analysis. The number of pairs included in the final analysis for each measure and for each movie is the following: Positive movie: N_EDA = 16; N_HR = 22; Negative movie: N_EDA = 18; N_HR = 21;

### Reactivity scores

Physiological reactivity for both emotional movies and for both physiological measures were calculated by computing delta scores between average scores of the emotional movie and average scores of the correspondent neutral movie.

### Inter-subject correlation analysis

The central result of this study was derived from the inter-subject correlation analysis (ISC). The ISC assesses the degree to which the response time-courses of two individuals co-vary, while taking into account lags between the responses. ISC (*C*_*xy*_) was defined as the maximal correlation within ±10 sec lags, where x and y are two individual, z-normalized response time-courses (see [41,42] for a similar approach in the autonomic domain and [43] in the neural domain). We quantified the experimental ISCs as $C_{x_{j}y_{j}}$, where *x*_*j*_ and *y*_*j*_ are autonomic response time-courses of two participants who saw the movie together, in the same group *j*. As a control, we quantified all pairwise ISCs for participants who saw the same movie in different groups, i.e. did not share a physical presence with each other. We hypothesized that participants who shared both the input from the movies and the physical presence with each other (experimental) would exhibit higher levels of ISC relative to those who only shared the input (control). This difference would in turn mark the physiological effect of co-presence (Fig 1 illustrates this approach).

To assess the statistical likelihood of the control ISCs we compared it to ISC computed on the surrogate data that preserves the temporal and the spectral characteristics of the experimental time series, applying nonparametric bootstrapping procedure on ([42] for details).

To assess the effect of co-presence (experimental ISC) we assumed that the control ISCs can serve as the null distribution for the H_1_ hypothesis, since all the experimental characteristics and the signal properties were identical in the experimental and control conditions. Accordingly, we applied a bootstrapping with repetition procedure on control ISCs to construct the control sampling distribution. The statistical likelihood of the experimental group-wise ISC was assessed directly from this control distribution. This procedure was done separately for each autonomic measure (yielding ISC_HR_ and ISC_EDA_) and for each emotional movie.

For the correlation analysis between ISC indexes and various behavioral measures, we computed a composite ISC score (ISC_composite_) by averaging fisher-transformed, z-normalized ISChr and ISCeda scores. Since ISChr and ISCeda were highly interrelated (movie one: r = 0.77, p<0.00; movie two: r = 0.44, p<0.03), the composite score provides a reliable estimate of a pair’s physiological coupling. The composite score was calculated for all dyads in the sample, for each emotional movie separately. To enhance the number of data points in our sample, for pairs missing either ISC_HR_ or ISC_EDA_, the composite score was comprised of the available ISC score. This procedure yielded 23 and 22 data points (dyads) in the positive and negative movie conditions respectively.

To assess the relationship between the ISC_composite_ scores and behavioral measures we applied non-parametric, Spearmen rank correlations tests. Applying Pearson correlations showed similar results.

The physiological and the behavioral measures collected in this study are provided in S1 Dataset.

## Results

### Positive and negative movies elicited distinctive emotional responses

The two emotional movies elicited differential emotional states: the strongest emotion elicited by the positive movie was joy (M = 5.4, SD = 1.67), and by the negative movie was fear (M = 6.7, SD = 1.5), which were higher than all other emotion scales (all p<0.001). In addition, a series of paired t-tests showed that compared to the positive movie, the negative movie was more arousing (t(25) = 3.54, p<0.002) and elicited higher physiological reactivity (EDA: t(15) = 2.75, p<0.01, RSA: t(20) = 1.81, p<0.08).

### Autonomic signals of co-present participants exhibit enhanced synchronization

We quantified ANS response synchronization as pairwise inter-subject correlation (ISC) of two individual ANS time-courses (see Methods for details). We quantified ISC for pairs of participants who saw the movie together (hereafter, experimental ISC) and for all possible pairs of participants who saw the same movie but in different groups (hereafter, control ISC). The control ISCs manifest movie-driven effects on ANS, that is the degree to which emotional movies elicited consistent ANS response time-course, which were similar across all participants [42]. We hypothesized that the ANS signals of the co-present participants will be more similar (i.e. higher ISC) than the ANS signals of participants who saw the movie in different groups.

**Fig 2:**
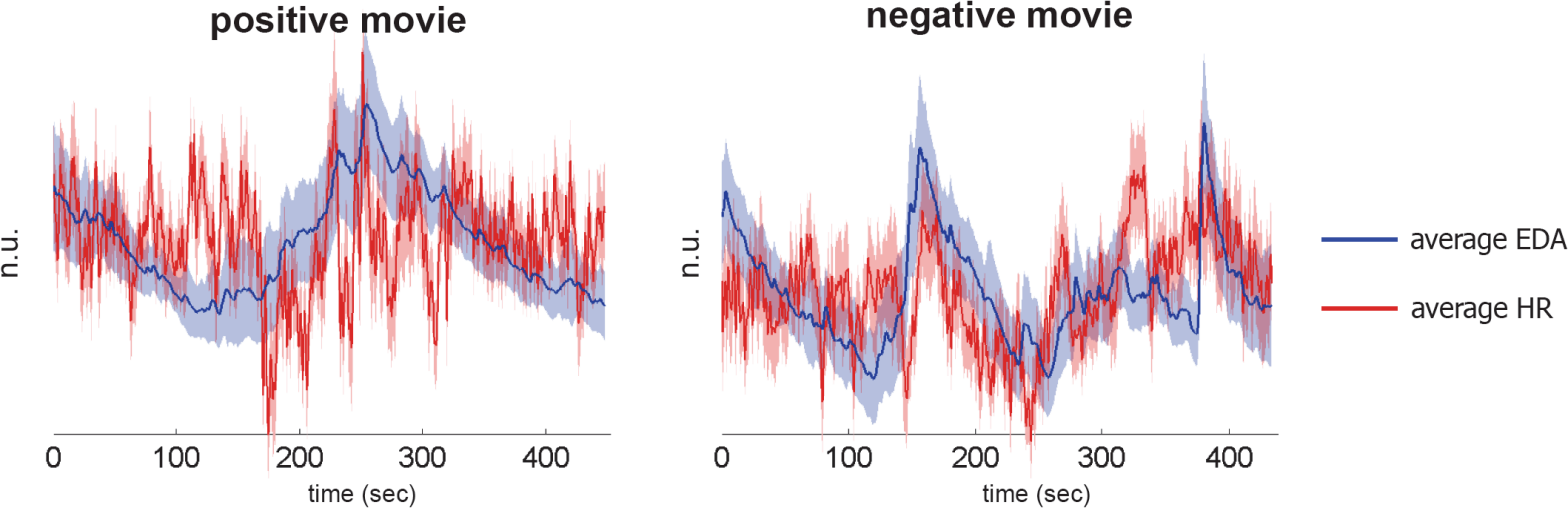
Average autonomic response time-courses elicited by positive and negative movies. Figure presents normalized EDA (blue) and HR (red) response time-courses (2HZ), averaged across participants. Shaded areas represent standard errors.

First, similar to our previous study[42], we found that emotional movies elicited consistent autonomic response patterns. Fig 2 presents average ANS signals elicited by positive and negative movies. Assessing response similarity across participants showed that the control ISCs were significantly larger than zero (p<0.01 for all four ISC measures, Table 1), indicating reliable movie-driven effects.

**Table 1:** Experimental and control ISCs.

	positive movie			negative movie		
	control	experimental	*p*	control	experimental	*p*
ISChr	0.1± 0.017	0.14; [0.13, 0.15]	p<0.01	0.14± 0.029	0.19; [0.15, 0.23]	p<0.03
ISCeda	0.36± 0.07	0.49; [0.37 0.63]	p<0.05	0.26± 0.07	0.44; [0.31, 0.56]	p<0.001

Descriptive statistics of the control sampling distribution (means and standard deviations) and the experimental sample (means and 95% CIs), as well as the p values for H_1_ hypothesis, computed for both autonomic measures (ISCeda, ISChr) and for both emotional movies (positive movie, negative movie).

**Fig 3:**
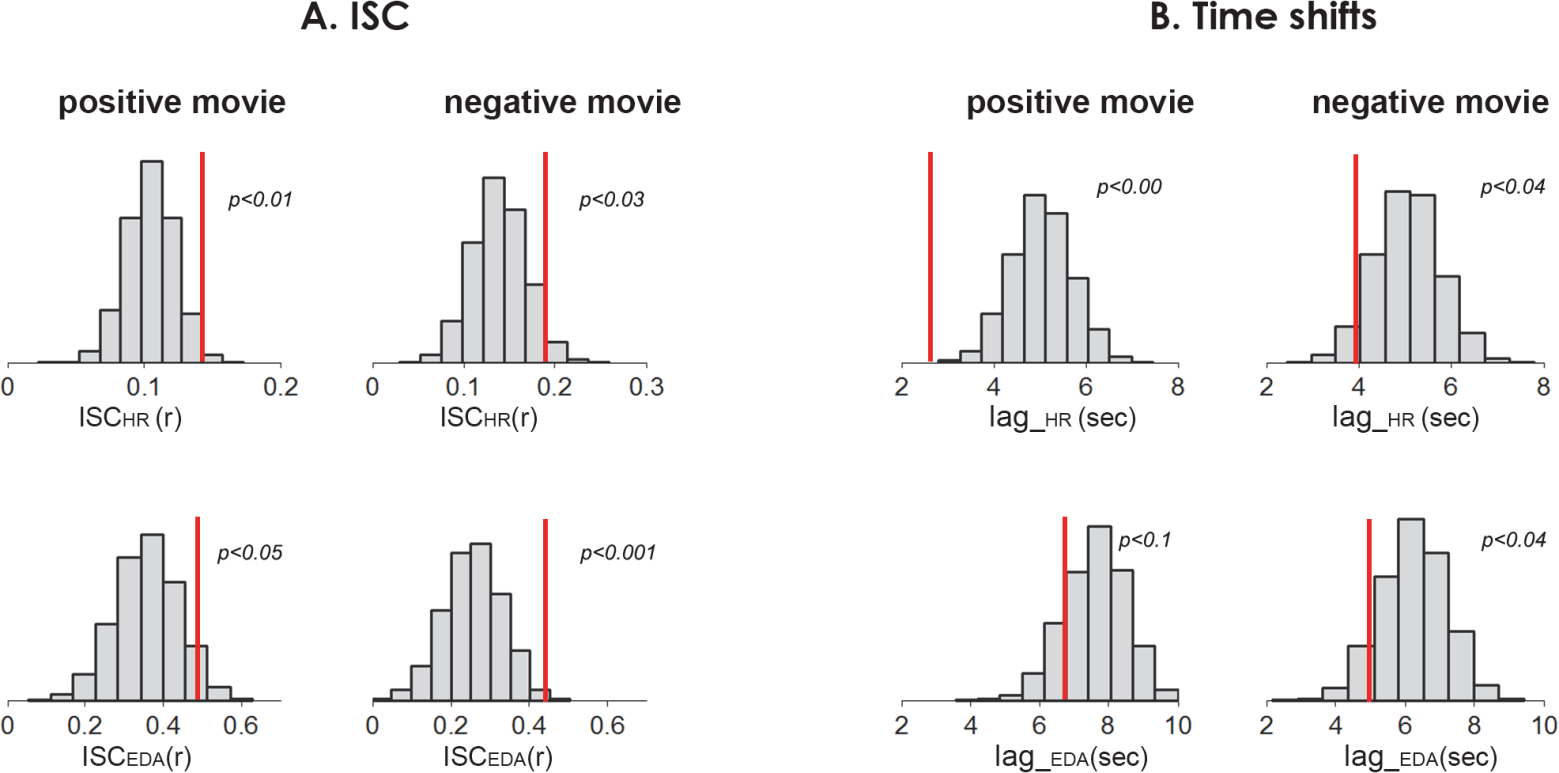
The effect of co-presence on autonomic ISC. **A.** Control (H_0_) distributions (not co-present) and the experimental means (co-present, dashed lines), for both autonomic measures (ISC_EDA_, ISC_HR_) and for both emotional movies. H_0_ distributions represent movie-driven ISC of ANS signals. As can be seen in the figure, the experimental group-wise ISCs fall in the right tail of the control distributions, indicating the positive effect of co-presence on synchronization of participants’ autonomic activity. **B.** Time shifts in ANS synchronization: we assessed whether the ANS signals of co-present individuals were more tightly linked in time. Figure presents the control (H_0_) distributions and the experimental means (dashed lines) of the time lags in which two ANS signals exhibited maximal ISC. As can be seen in the figure, the time shifts in ANS synchronization of the co-present participants were significantly smaller in three out of four ANS measures. Lag_EDA in the positive movie showed a trend towards significance.

In accordance with our hypothesize, the experimental ISCs were larger than the control ISCs, indicating that the response time-courses of co-present participants were idiosyncratically aligned with each other, above and beyond the effects induced by the movie. The statistical likelihood of the co-presence effect (experimental ISCs) was directly estimated from the sampling distribution of control ISCs, applying a non-parametric procedure, for both autonomic measures and for both emotional movies (Fig 3A, see Methods for details). The descriptive statistics for the control sampling distribution and the experimental sample are displayed in Table 1.

To account for possible nonstationarity effects in physiological data, we also performed the ISC analysis in consecutive temporal windows of 60 seconds. ISC scores were computed for each temporal window and then averaged across windows. Nonparametric analysis of the windowed ISC scores, averaged across time, (wISC), further supported the co-presence effect on autonomic synchronization, showing increased wISC in the experimental as compared to control condition (see S1 Fig for wISC time-courses and wISC distributions).

Finally, we examined the temporal differences between the movie-driven response synchronization (control ISC) and response synchronization associated with co-presence (experimental ISC). For that aim we assessed the time shifts at which two individual time-courses exhibited maximal correlation in the experimental and the control conditions. This analysis showed that the ANS time courses in experimental condition were more tightly linked (shorter lags) than in the control condition. Fig 3B presents the experimental mean and the control sampling distribution for the temporal lag analysis. The descriptive statistics for this analysis are displayed in Table 2.

**Table 2:** Experimental and control time-shifts.

	positive movie			negative movie		
	control	experimental	*p*	control	experimental	*p*
lag_HR (sec)	5.05± 0.65	2.7± 0.44	p<0.00	5.05± 0.69	3.9± 0.64	p<0.04
lag_EDA (sec)	8.1± 0.8	7.09± 0.8	p<0.1	6.2± 0.9	4.9± 0.82	p<0.04

Descriptive statistics of the control sampling distribution (means and standard deviations) and the experimental sample (means and standard error), as well as the p values for the H_1_ hypothesis, computed for both autonomic measures (lag_EDA, lag_HR) and for both emotional movies (positive movie, negative movie).

Taken together, these results suggest that while the emotional movies induced similar responses across all study participants, the participants who saw the movie together underwent additional mutual processes. In particular, the ANS time-courses of the co-present participants were more similar in their dynamics (Fig 3A) and more tightly linked in time (Fig 3B), in comparison to control condition.

### Autonomic synchronization across co-present participants was associated with the convergence of their emotional responses

To investigate the relation between physiological synchronization and shared affect during co-presence, we first assessed whether co-present participants indeed showed convergence in the emotions elicited by the movie. To that aim we computed intra-class correlation coefficients (ICCs) for the ratings of joy in the positive movie and ratings of fear in the negative movie. We found moderate, marginally significant ICC for positive movie (r = 0.3, p<0.06, two way random model) and high, significant ICC for negative movie (r = 0.613; p<0.00, two way random model).

**Fig 4:**
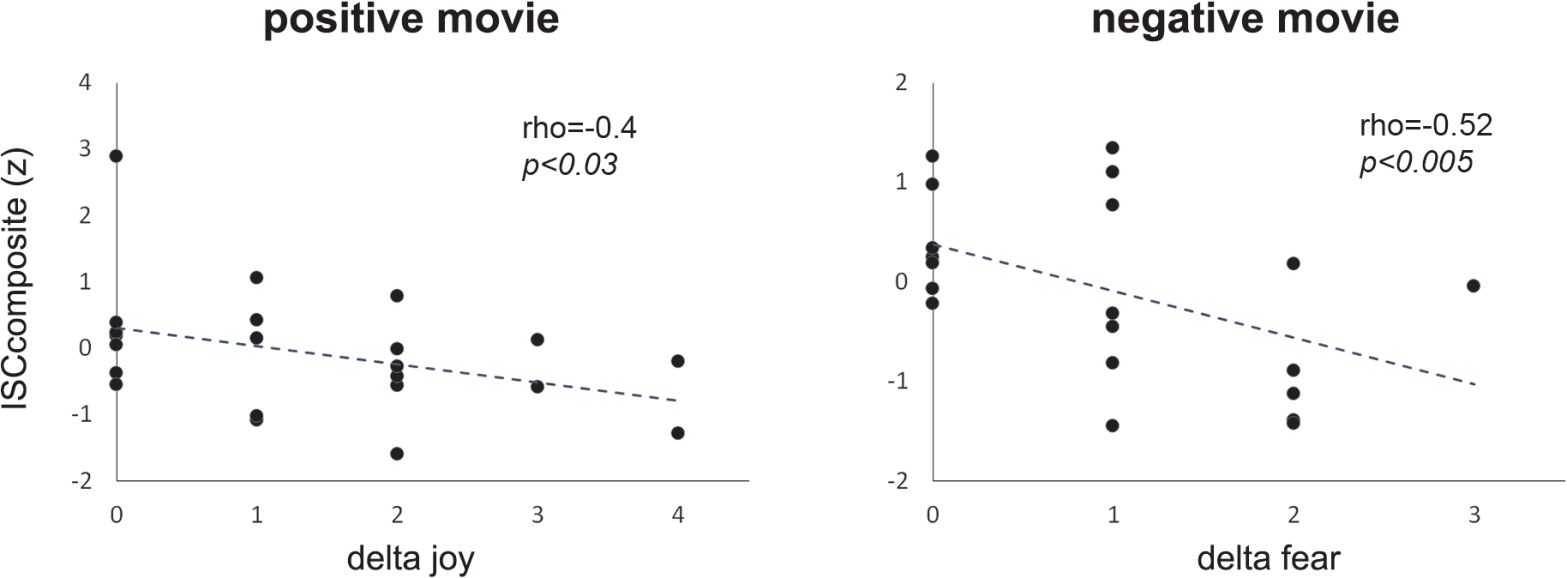
Association of autonomic synchronization and emotional convergence. Correlation between the extent of autonomic synchronization, as indexed by ISC_composite_ scores, and the degree of emotional convergence (small delta scores signify higher convergence). Results are shown separately for positive and negative emotional movies.

We then calculated the emotional convergence scores as the absolute difference (delta) between the emotion ratings of pair members (note that a small delta signifies higher convergence) and assessed its relationship with the ISC scores (as indexed by ISC_composite_: the average of the normalized ISC_EDA_ and ISC_HR_ scores for each pair (see Methods)). In accordance with our hypothesis, we found that ISC_composite_ scores were directly associated with the emotional convergence scores (Fig 4; positive movie: rho = -0.4, p<0.03; negative movie rho = -0.52, p<0.005; Spearman rank correlation, one tailed).

We investigated the possible sources of the inter-pair variability in emotional convergence and physiological coupling. First, we assessed whether the emotional and the physiological coupling indexes were associated with the reported emotional intensity of the movies. For both movies, emotional convergence was moderately (not reaching significance) correlated with the arousal scores (r = -0.34, p<0.09; r = -0.27, p<0.17, for positive and negative movies respectively) and the valence scores (r = -0.3, p<0.14; r = 0.3, p<0.07; for positive and negative movies respectively). The ISC_composite_ scores were not correlated with any of these measures (all p>0.5).

The participants in the current study were acquaintances. Previous studies showed that friends are more similar than strangers in their emotional responses [37,44]. Hence, we assessed whether the variability in the emotional and the physiological convergence of participants can be explained by their level of acquaintance. The degree of acquaintance was negatively related to the degree of emotional convergence in both movies (positive movie: r = 0.62, p<0.001; negative movie: r = 0.47, p<0.016) and negatively correlated with the ISC scores in the positive movie (r = -0.4, p<0.05), while showing no relationship in the negative movie (r = -0.01, p = 0.9). The friendship scores, reported by the participants, ranged from 2.5 to 9.5 with a mean = 5.7 and std = 2.13, suggesting that on average the participants were school acquaintances and not close friends, which might explain the inconsistency with the previous findings. These results are important, as they disprove the possibility that physiological and emotional coupling in our study can be explained by the initially more similar responses to movies among more familiar participants.

## Discussion

In this study we investigated whether “merely co-present”, not communicating individuals can unintentionally shape each other’s states, exhibiting temporally synchronized autonomic responses and convergent emotional experiences. We found that co-present participants who viewed emotional movies together reported similar emotional responses to the movies. Importantly, we also found that the autonomic signals of these participants were distinctively synchronized, above and beyond the movie-driven synchronization. To the best of our knowledge, this is the first demonstration that spontaneous autonomic coupling in emotional context can arise in a non-communicative situation, when individuals’ attention is directed toward an external stimulus.

In real-life people frequently act and respond in close proximity with others—e.g., viewing movies in the cinema, working side-by-side with other people, observing a person in need while walking with a group of friends. The effects of the mere presence of others on one’s own responses have been studied in the past in social psychology, demonstrating general arousing effects, such as facilitation of the dominant response in cognitive and behavioral tasks [45] as well as enhancement of facial emotional expressions [46]. More recent research has begun to unveil how the merely present others distinctively modify one’s own responses. For example, a series of studies showed that actions and goals of another, co-present individual are automatically co-represented and can interfere with one’s own performance [47,48]. This accumulating body of work suggests that the mere presence of another individual automatically activates the mechanisms targeted to share information with others [32]. Our results support this framework by demonstrating that even in a minimally social set-up, individuals unintentionally affect each other’s states, exhibiting similar autonomic and emotional responses.

What is the neurobiological mechanism that drives autonomic synchronization across individuals in social emotional contexts? The results of the current study suggest two different sources for such interpersonal ANS alignment. First, emotional movies induced consistent, temporally locked emotional events in the observers. Accordingly, the ANS signals across all participants in the study were similar (represented by control ISCs). The control ISC thus manifests the dynamic alignment of ANS activity with the emotional input, coming from the movies. Similar results were demonstrated in previous studies, showing that naturalistic movies elicit consistent response patterns in autonomic and brain signals, which become synchronized across individuals [42,49].

Besides the movie-driven component, shared across all the participants, the co-present individuals exhibited an additional, idiosyncratically shared ANS variability. That is, the ANS signals of the co-present participants were more similar in their dynamics and were more tightly coupled in time (Fig 3). We suggest that this dyad-specific synchronization is a result of recursive interpersonal influences, during which individual differences in the intensity and the dynamics of the emotional events were propagated across the co-present viewers, leading to shared emotional experiences. In support of this interpretation, the degree of autonomic synchronization between the co-present participants was correlated with the level of their emotional convergence. While the correlational nature of this result cannot allow for the establishment of the causal route between interpersonal autonomic coupling and shared emotional experiences, it provides evidence that these are tightly linked.

To summarize, while all the participants in the study were synchronized with the emotions elicited by the movie, the co-present ones were also synchronized with the emotional signals of each other. Previous studies have demonstrated that the emotions of other people elicit corresponding emotional and physiological states in the observers [3,7,9]. We extend these studies by showing that the contagious spread of emotional signals can arise unintentionally in minimal social conditions, and that these transmissions are manifested in the dynamics of the ANS signals.

The sensory channels through which transmission of emotional information can occur in non-interactive social conditions is an important question, warranting future research. Recent research demonstrating emotional detection in visual periphery [50] suggests that subtle peripheral cues (e.g. facial and postural emotional signals) from the other individuals can be traced and incorporated into one’s own emotional state. In addition, contagious emotional effects in minimal social contexts can be mediated by chemosignals [51], which do not necessitate conscious allocation of attention.

Although we found that at the group level individuals significantly affected each other's emotional and autonomic responses, there was a large variability among pairs as to the degree of this effect. While previous studies have found that interpersonal characteristics, such as the type and quality of the relationship can influence autonomic [23,24,29] and emotional [37,44] convergence, we didn’t find such effects in our sample. Additional research is needed to unveil the personal and interpersonal factors affecting the degree to which individuals are influenced by the emotional responses of others. Notably, the mere co-presence setups, such as the one used in this study, are beneficial for isolating such factors, as they circumvent the complexities of direct interaction.

## Conclusions

In conclusion, this study demonstrates that co-present individuals can shape each other's autonomic and emotional responses and become synchronized in the absence of direct, face-to-face communication. In addition, the results of the current study indicate that synchronization of ANS signals across individuals in social emotional context is related to the emergence of shared emotional experiences. Future research is needed to establish whether ANS synchronization has a direct casual role in contagious emotional processes between people.

## Supporting Information

S1 Dataset A. Behavioral responses. Dataset includes SPSS file with behavioral ratings of both movies and both observers. **B.** Autonomic signals. Matlab file with ANS signals of both observers. “signals.mat” contains 2HZ physiological time-courses (EDA and HR) for 26 pairs of subjects in four experimental conditions. Noisy time-courses that were excluded from analysis are replaced with NaNs. 1x4x2 struct array: 2^nd^ dim – 4 experimental conditions: 1- baseline, 2 – positive movie, 3- negative movie, 4 – first neutral movie; 3^d^ dim – signals: 1—HR, 2 – EDA;
(ZIP)

S1 Fig ISC in short temporal windows. A) Dynamics of windowed ISC (wISC) in experimental (red) and control (blue) conditions, computed for each ANS measure (HR –upper row, EDA- lower row), and for each emotional movie. wISC is computed in 60 sec windows with 30 sec overlap. B) To assess the effect of co-presence on wISC, we averaged the wISCs across non-overlapping windows, both for co-present dyads (experimental wISCs) and for control pairs from different groups (control wISCs). Figure presents the control sampling distributions and the experimental means of the average wISC, calculated for both emotional movies and for each ANS measure. As can be seen in the figure, this analysis supports the main results of the study, exhibiting enhanced wISC in the experimental as compared to control conditions. Specifically, three out of four wISC measures exhibit significance (p<0.01), with one measure showing trend for significance (p<0.1).
(TIF)
