# Supplementary figures and images for: The Mere Co-Presence: Synchronization of Autonomic Signals and Emotional Responses across Co-Present Individuals Not Engaged in Direct Interaction
Source: PLoS One. 2015 May 27;10(5):e0125804. doi: 10.1371/journal.pone.0125804 (PMC4446307; doi:10.1371/journal.pone.0125804)

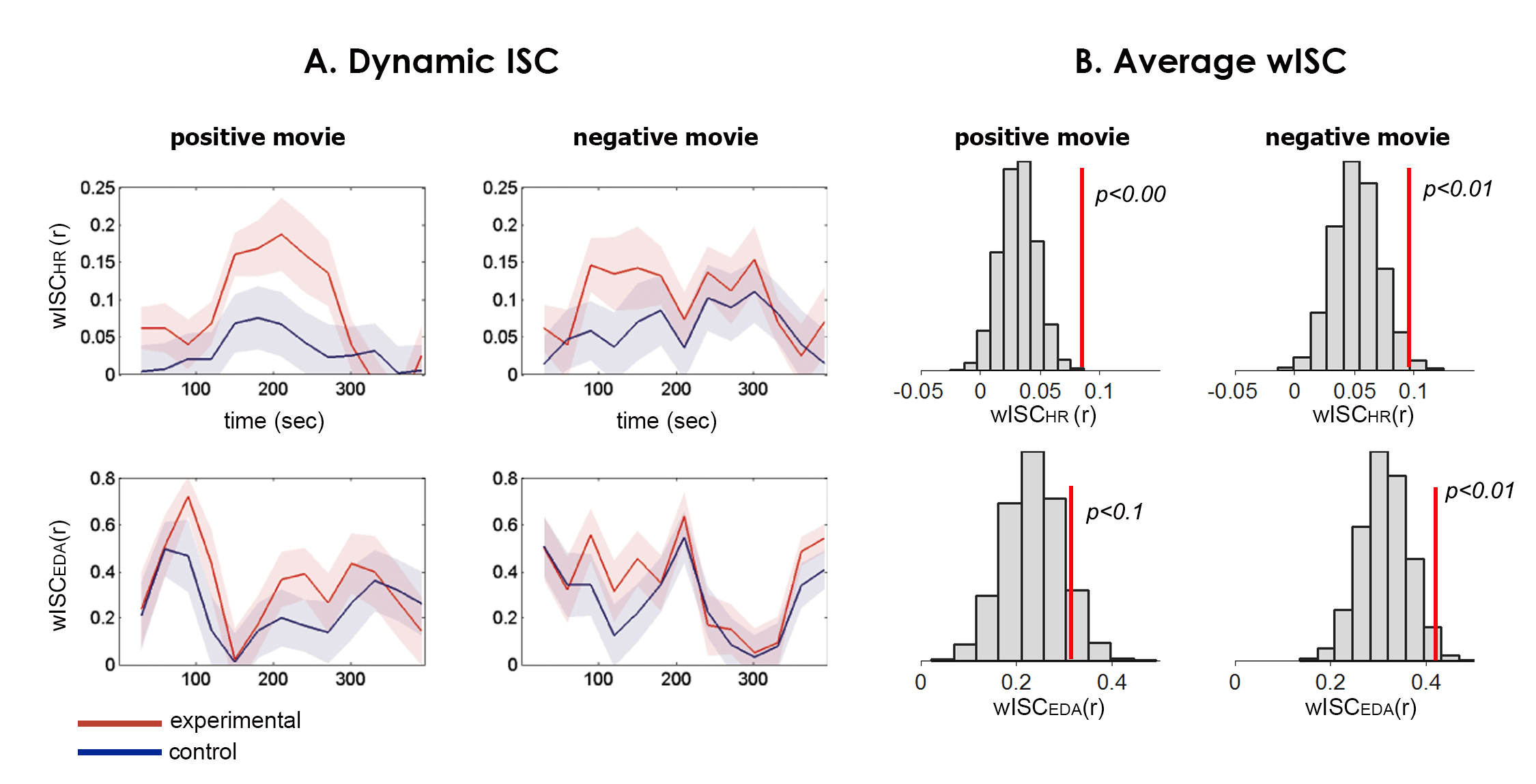

Supplement: S1 Fig — A) Dynamics of windowed ISC (wISC) in experimental (red) and control (blue) conditions, computed for each ANS measure (HR –upper row, EDA- lower row), and for each emotional movie. wISC is computed in 60 sec windows with 30 sec overlap. B) To assess the effect of co-presence on wISC, we averaged the wISCs across non-overlapping windows, both for co-present dyads (experimental wISCs) and for control pairs from different groups (control wISCs). Figure presents the control sampling distributions and the experimental means of the average wISC, calculated for both emotional movies and for each ANS measure. As can be seen in the figure, this analysis supports the main results of the study, exhibiting enhanced wISC in the experimental as compared to control conditions. Specifically, three out of four wISC measures exhibit significance (p<0.01), with one measure showing trend for significance (p<0.1). (TIF) [file pone.0125804.s002.tif]
